# Early Detection, Diagnosis, Prevention, and Treatment of Infection to Avoid Sepsis and Septic Shock in Severely Burned Patients: A Narrative Review

**DOI:** 10.3390/ebj6010006

**Published:** 2025-02-06

**Authors:** Patrick M. Honoré, Sydney Blackman, Emily Perriens, Jean-Charles de Schoutheete, Serge Jennes

**Affiliations:** 1 CHU UCL Namur Godinne, UCL University, Campus Godinne, 5530 Yvoir, Belgium; 2 Faculty of Medicine, Experimental Research Laboratory Institute of the Catholic Louvain Medical School, 1200 Brussels, Belgium; 3 Chirec Hospitals, ULB University, 1050 Brussels, Belgium; blackman.sydney@gmail.com; 4 Faculty of Medicine, ULB University, 1050 Brussels, Belgium; perriens.emily@gmail.com; 5 Burn Unit, Grand Hopital de Charleroi, 6000 Charleroi, Belgiumserge.jennes@ghdc.be (S.J.)

**Keywords:** MeSH burns, early diagnosis, primary prevention, therapeutics

## Abstract

**Highlights:**

**What are the main findings?**

- Plasma levels of the N-terminal fragment of the pro-hormone brain-type natriuretic peptide forecast more accurately the development of early infection and sepsis than procalcitonin in cases of grave burned. Revolutionary new biomarkers include the use of polymerase chain reaction (PCR) and gene expression (mRNA) profiling to gain a diagnostic advantage over current methodologies, allowing detection as early as 4 to 6 h after intensive care unit admission. Mass spectrometry is a revolutionary recent tool for the rapid determination of bacteria, yeast, and fungi based on their bacteria protein profiles.
- Unfortunately, these tools are not readily available at the bedside in most burn centers, leaving burn intensivists to rely primarily on clinical criteria and older biomarkers (C-reactive protein and procalcitonin), likely due to cost considerations.

**What is the implication of the main finding?**

- The early detection, diagnosis, anticipation, and therapy of infection to avoid sepsis and septic shock remain significant challenges in severely burned patients. The limited number of burn centers worldwide makes it difficult to conduct randomized controlled studies that are crucial for improving the early detection, prevention, and treatment of infections to prevent sepsis and septic shock. Large congresses on burn infections are urgently needed to gather a majority of burn intensivists from around the world to launch these essential randomized controlled studies.

**Abstract:**

The early detection, diagnosis, anticipation, and therapy of infections to prevent sepsis and septic shock remain significant challenges in cases of grave burns. This narrative review explores various tools for early infection detection, including emerging biomarkers, the American Burn Association’s clinical criteria, and traditional blood parameters. A comparative study of the American Burn Association, Mann-Salinas, and Sepsis-3 criteria highlights the superior early detection capabilities of the Sepsis-3 criteria. However, the authors recommend that sepsis should be prospectively evaluated, identified, and classified by the intensive care group, rather than by relying solely on retrospective items, though the latter may still be necessary in certain cases. Advances in biomarker identification, including polymerase chain reaction (PCR) and gene expression (mRNA) profiling, offer diagnostic advantages over current methods, enabling early detection within 4 to 6 h of intensive care unit admission. Mass spectrometry also shows promise for the rapid determination of bacteria, yeast, and fungi based on bacteria protein profiles. Source control remains crucial, and the use of antibacterial topical agents has significantly improved the survival rates of severely burned patients. However, antibiotic selection must be made judiciously to avoid resistance. Despite these advancements, significant progress is still needed to improve the rapid identification, actual presence, prevention, and therapy of infections to reduce the incidence of sepsis and septic shock in this patient subgroup.

## 1. Background

Diagnosing infection and sepsis in cases of grave burns continues to be an important hurdle [1]. Burn injuries are inherently linked to states of inflammation, which play a dual role in the recovery process [1]. Acute-phase immune activation is crucial for survival and wound healing, but inflammation, while protective against infection, can become detrimental when triggered by concurrent infections or conditions such as sepsis or pancreatitis. Differentiating between inflammation caused by burns and that resulting from infections remains a common clinical dilemma.

To address this challenge, biomarkers have increasingly been utilized to enable faster treatment initiation, especially prior to the confirmation of blood cultures—although this approach tends to be more effective for fungal infections than bacterial ones.

Despite their lack of specificity, indicators like C-reactive protein (CRP) and white blood cell (WBC) count are continuously used. However, more recently, procalcitonin (PCT) was designated as a better identifier for diagnosing and predicting infections in cases of grave burns [2]. The surveillance of PCT levels for a prolonged period can help guide antibiotic therapy, reduce its use beyond what is necessary, and minimize the danger of producing multidrug-resistant bacteria. Nevertheless, due to limited opportunity and high costs, PCT has not yet replaced CRP in the hands of physicians. Additionally, recent prospective trials have questioned the benefit of PCT and suggested other promising indicators for diagnosing sepsis in burn cases [1,2].

Beyond biomarkers, this review will examine other diagnostic tools, including advances in PCR and gene expression (mRNA) profiling, which offer superior diagnostic capabilities, allowing for the detection of infections as early as 4 to 6 h after intensive care unit (ICU) admission. This review will also discuss infection prevention measures specific to burn units and strategies for early infection treatment. Expensive therapies, such as bacteriophage treatments, will be considered as well. Despite recent advances, significant progress remains to be made in improving the early detection, diagnosis, prevention, and treatment of infections to prevent sepsis and septic shock in severely burned patients.

## 2. Proposed Algorithm to Differentiate Colonization from Deep Skin Infection

In our view, the first step is to diagnose deep skin infections and to differentiate colonization with help of clinical labs and bacteriology, and with the help of biologic biomarkers of inflammation-infection afterwards.

### Factors Favoring Deep Skin Infections (In Order of Importance)

(1) Positivity of bacteremia (although the lack of bacteremia does not eliminate the presence of sepsis).
(2) Positivity of (massive inflammation) criteria.
(3) Presence of sepsis or septic shock.
(4) Rapid and significant elevation of biomarkers within 12 h.
(5) Sharp and significant elevation of liver enzymes within 12 h.
(6) Rapid and marked elevation of leukocytosis within 12 h, accompanied by evidence of disseminated intravascular coagulation (DIC).
(7) Recent quantitative positive cultures from a central or arterial line (compared with blood cultures through a venous puncture), showing that the infection is linked to a catheter rather than a deep skin infection.

The absence of these six criteria makes the diagnosis of deep skin infections unlikely.

This algorithm is a proposal of our group and is the result of our reflections within our burn unit. This has been modified quantitatively from the Summary of the 2012 ABA Burn Quality Consensus Conference [3]

## 3. Review—Core Text

### 3.1. Detection of Early Infection

#### 3.1.1. Use of Bacterial and Fungal Biomarkers to Detect Early Infection

More recently, as CRP and PCT proved disappointing, Paratz et al. [4] identified that plasma levels of the N-terminal fragment of the pro-hormone brain-type natriuretic peptide (NT-proBNP) predicted early infection and sepsis in severely burned patients better than PCT. However, this finding was not corroborated by a recent meta-analysis [5]. Similarly, Cakır Madenci et al. [6] mention that the soluble CD14 subtype (presepsin) demonstrated identical positivity of the test and prognosis evaluation to that of CRP and PCT. Nonetheless, their study faced criticism due to the observed significant differences in presepsin, CRP, and WBC counts, but not in PCT levels, in the first 24 h of sepsis in contrast with previous days.

In a recent randomized controlled trial (RCT) evaluating the therapeutic ability of blood purification (BP) in cases of severe burns with sepsis, researchers assessed the prognosis of serum values of PCT, CRP, and BNP [7]. The trial identified no substantial differences in serum PCT and CRP values between survivors and non-survivors (*p* > 0.05). However, serum BNP values were substantially lower in survivors than in those who succumbed (*p* < 0.05). Receiver operating characteristic (ROC) curve evaluation revealed a low predictive level for PCT and CRP in burn sepsis prognostic value, while BNP showed good predictive value (*p* < 0.05). Early BP treatment was shown to substantially ameliorate the prognostic value of burn sepsis, though the more frequent use of BP in survivors may have artificially lowered BNP levels. Nonetheless, the lack of difference in PCT levels between groups suggests that BNP may be a more favorable identifier for the quicker diagnosis of infection and sepsis in this RCT of 119 patients [7].

Looking at other trials, a recent meta-analysis of 10 trials involving 704 cases looking at PCT as a biomarker demonstrated associated sensitivity, specificity, positive likelihood ratios, negative likelihood ratios, and diagnostic odds ratios of 0.67 and 13.70 [8]. The area under the summary receiver operating characteristic curve (SROC) was 0.85 (95% CI: 0.82–0.88), with the diagnosis level as the main origin of heterogeneity. These findings put forward the idea that serum PCT can be seen as an interesting identifier for the quicker diagnosis of burn sepsis in adults and could be associated with different positivity markers to enhance sensitivity and specificity [8].

The challenge of identifying a more effective identifier for quicker infection and sepsis detection in cases of severe burns remains unresolved. While CRP and WBC count are still the most commonly used markers [8], the reliability of PCT as a positive testing tool in burn cases remains uncertain [9]. Recently, consensus definitions for sepsis and infection have been introduced, specifically tailored to burn populations. These definitions are frequently used in clinical practice, though they still require further validation [10].

At present, there is no clear consensus on the optimal biomarker for this patient group. Combining multiple biomarkers may offer a more effective diagnostic approach, but further research and more robust data are necessary to support this strategy [4]. Additionally, the timing of both sample collection and detection display pivotal insights into the early diagnostic process, particularly when analyzing different types of samples, such as burn tissues, pus, and secretions [10,11,12,13,14]. Plasma levels of the N-terminal fragment of the pro-hormone brain-type natriuretic peptide are a better predictor of early infection and sepsis development than PCT. Although there are some new detection methods in the clinic, they are still not popular in clinical application. Burn physicians still rely on clinical criteria and older biomarkers (CRP and PCT).

Regarding fungal biomarkers, although the serum 1,3-beta-D-glucan test has been used as an early diagnostic marker of candidemia, there are few studies regarding the association of serum 1,3-beta-D-glucan levels with candidemia in severe burn patients [12].

#### 3.1.2. American Burn Association Consensus Panel Publication on Criteria to Detect Early Infection in Severe Burn Cases, Including a Critical Review of the Criteria

The diagnosis of early infection in severely burned patients is complex and challenging. The American Burn Association (ABA) has delivered consensus criteria to aid clinicians in identifying invasive and noninvasive burn wound infections [12,13]. This section presents these criteria along with a critical analysis of their applicability and limitations.

1. **Invasive Burn Wound Infection**

Invasive burn wound infection is defined as an infection that occurs in deep partial-thickness or full-thickness burn wounds. It is typically combined with changes in the aspect or nature of a burn wound, such as quick eschar dislocation or dark brown, black, or violaceous disfigurement. These infections require debridement by a surgeon of the affected tissue and intravenous antibiotic treatment. Additionally, invasive burn wound infections are possibly accompanied by one or more of the following signs:

- **Inflammation:** Edema, erythema, warmth, or tenderness in the vicinity of normal skin.
- **Histopathologic Evidence**: Invasion of the infectious bacteria in the vicinity of normal tissue observed in a burn biopsy specimen.
- **Positive Blood Culture**: Bacterial growth in a blood specimen with a lack of auxiliary recognizable infections.
- **Global Signals of Infection**: Hyperthermia, hypothermia, leukocytosis, tachypnea, hypotension, oliguria, hyperglycemia at a formerly authorized value of dietary carbohydrate, or mental disorientation.

The criteria for invasive burn wound infection, although useful, are not without limitations. Changes in the appearance of the wound or eschar, while indicative of infection, are not specific enough to be conclusive, as they may be due to other factors such as wound progression or inadequate perfusion. Furthermore, while inflammation in surrounding uninjured skin can be a sign of infection, it can appear as a consequence of other non-infectious diseases. Histopathology, though valuable, is not always feasible due to resource limitations in burn centers. Finally, systemic signs of infection can be nonspecific and may mimic other conditions, such as pulmonary embolism or widespread inflammation unrelated to infection.

2. **Noninvasive (Local) Burn Wound Infection**

Noninvasive burn wound infections are more commonly observed and typically occur in healing partial-thickness burns or grafted full-thickness wounds. These infections can induce postponed wound healing or loss of skin grafts. According to the ABA [3], a noninvasive infection is indicated by the presence of purulent exudate, which may be culture-positive (if cultures are obtained), along with the need for a change in treatment. This may include altering antimicrobial therapy, removing wound coverings, or increasing the frequency of dressing changes. Furthermore, one or more of the following signs may be present:

- **Loss of Wound Covering**: Loss of synthetic or biologic coverings of the wound.
- **Changes in Wounds**: Aspect like hyperemia.
- **Erythema**: In the normal skin in the vicinity of the wound.
- **Global Signals:** Hyperthermia or leukocytosis.

While these criteria are helpful in diagnosing noninvasive infections, they also have limitations. A positive culture from purulent exudate may not distinguish between true infection and bacterial colonization, as wounds are frequently colonized by bacteria without necessarily being infected. Additionally, non-specific signs such as the loss of wound covering, erythema, and systemic inflammatory responses like hyperthermia or leukocytosis can result from a variety of factors and are not definitive indicators of infection.

3. **Microorganisms Responsible for Burn Wound Infections**

Burn wound infections can be induced by a variety of microorganisms, including bacteria, fungi, and viruses. Bacterial pathogens include both Gram-positive organisms—like *Staphylococcus aureus*, β-hemolytic *Streptococcus* group A, and *Enterococcus* species (including vancomycin-resistant strains)—and Gram-negative organisms, such as *Pseudomonas aeruginosa*, *Acinetobacter baumannii*, and enteric organisms like *Klebsiella* species, *Escherichia coli*, or *Enterobacter* species. Fungal infections, particularly *Candida* species, are commensal flora or colonized but can become pathogenic. Environmental fungi, most commonly *Aspergillus* species, can lead to very severe infections and extensive tissue damage. Viral infections are typically caused by *Herpes simplex* virus, and are not so common.

These microorganisms are well recognized in burn wound infections, and we agree with the ABA’s identification of these pathogens as being significant contributors to the morbidity and mortality associated with burn injury lesions.

4. **Antimicrobial Resistance**

Antimicrobial resistance is an increasing concern in the treatment of burn wound infections, as it can severely limit treatment options and reduce the efficacy of both prevention and therapy engagements. Bacteria of particular worry comprise methicillin-resistant *Staphylococcus* aureus, vancomycin-resistant *Enterococcus*, and multidrug-resistant *Acinetobacter baumannii* and *Pseudomonas aeruginosa*. Some strains of Acinetobacter and Pseudomonas have developed resistance to nearly all tested antimicrobials, with the exception of colistin, which underscores the growing difficulty in treating these infections.

We concur with the ABA’s recognition of antimicrobial resistance as a critical issue, particularly with the increasing occurrence of multidrug-resistant organisms in burn patients, which poses significant challenges for treatment and outcomes.

5. **Limitations of the ABA Criteria and the Need for Additional Diagnostic Tools**

Using these criteria alone may not always be sufficient, and in some challenging cases it may be prudent to combine them with biomarkers [10]. The following section is based on the most recent consensus guidelines published in 2012 by the ABA for the diagnosis of burn infections [3].

A major limitation of the ABA criteria is that they are not always conclusive. Quick diagnostic methods like polymerase chain reaction (PCR) and mass spectrometry for bacterial and yeast identification could significantly improve the speed and accuracy of diagnosis [14]. However, these advanced methods are not widely available in all burn centers, limiting their access in daily clinical work.

6. **Recent Guidelines and Triggers for Considering Sepsis in Burn Patients**

The recent Surviving Sepsis After Burn campaign, outlined by Greenhalgh et al. (Burns, 2023), provides updated guidelines for diagnosing sepsis in burn patients. These guidelines were developed using rigorous methodologies, including PICO definitions and GRADE assessments [15]. The following are key triggers for considering sepsis in burn patients:

- **Change in SOFA ≥ 2 Points**: Indicates new or worsening organ dysfunction.
- **Lactate Level > 2 mmol/L (>18 mg/dL)**: Serves as a surrogate for base deficit and indicates hypoperfusion.
- **Temperature Modification:** New onset of fever or hypothermia (no consensus on threshold temperature).
- **Sudden Fall in Platelet Values**: Suggestive of coagulation abnormalities.
- **Decrease in Urine Output/Increased Fluid Requirements**: Indicates potential renal dysfunction.
- **Kidney Disease Improving Global Outcome (KDIGO) Acute Kidney Injury Stage ≥ 1**: Best practice statement.
- **Pulmonary:** Modifications such as increased respiratory rate or need for ventilator support.
- **Alterations in Mental Status**: Including confusion or decreased level of consciousness.
- **Gastrointestinal Dysfunction**: Signs like iléus or absent bowel sounds.
- **Changes in Wound Aspect Evocating Infection**: As previously described in the criteria.
- **Procalcitonin Rise ≥ 2 ng/mL from Initial** Value: May indicate bacterial infection.

Although these guidelines provide a comprehensive approach to sepsis diagnosis, they do not incorporate newer diagnostic tests, highlighting the continued need for accessible, rapid diagnostic tools in burn care settings.

#### 3.1.3. Other Classical Biomarkers in Routine Laboratory Analyses

**Figure 1:**
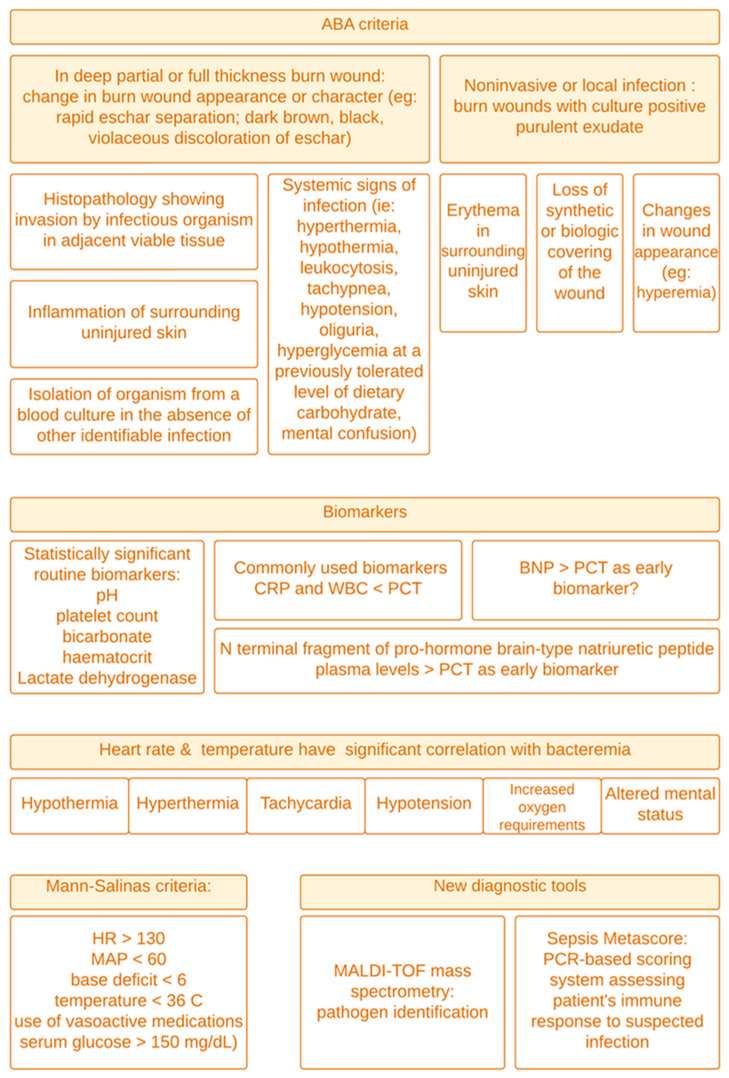
Identifying and evaluating potential diagnostic avenues for the early detection of infection or sepsis.

In a recent retrospective study [16], the authors demonstrated that pH, platelet count, bicarbonate, hematocrit, and lactate dehydrogenase (LDH) were classical identifiers that showed statistical significance in detecting early infection. A figure summarizing early detection is provided (Figure 1).

## 4. Diagnosis Sepsis

### 4.1. Comparison of Different Criteria to Diagnose Early Sepsis in Severely Burned Patients

Context: Sepsis is always difficult to diagnose in non-burn ICUs. The idea behind this section is to see if the criteria used for sepsis outside burn units are valid or not in burn units. Looking at a cohort trial of 418 patients, conducted from 2000 to 2016, 88 (21%) developed sepsis. The mean age of the cohort was 50 ± 18 years, with a mean total body surface area (TBSA) burn of 30% ± 17%. Inhalation injury was present in 50% of patients, the median duration of hospitalization was 49 days (interquartile range: 29–71 days), and the mortality rate was 19%. The ABA, Mann-Salinas [17], and Sepsis-3 criteria [18] were positive in 59%, 28%, and 85% of cases, respectively (*p* < 0.05). The best dependable predictor of sepsis included increased oxygen needs, confusion, hypothermia, hyperthermia, tachycardia, and hypotension. The authors concluded that the Sepsis-3 criteria were the most accurate predictors from non-burn units, followed by the ABA and Mann-Salinas criteria [3,17]. Nonetheless, no single criterion was sufficiently accurate to serve as a classical diagnostic in these burn cases. As a result, researchers recommend that sepsis be clinically assessed, diagnosed, and registered prospectively by a burn intensivist, rather than relying solely on retrospective criteria [19]. Nevertheless, it is acknowledged that retrospective criteria are often necessary in certain situations.

### 4.2. Comparison Between Burned Patients and Non-Burned Intensive Care Patients in the Diagnosis of Sepsis

The initial hypermetabolic and inflammatory reaction in burn patients results in many of the early assessment criteria—such as tachycardia, tachypnea, hypotension, fever, and leukocytosis—being almost nearly present when the cases were not infected. Accordingly, these scoring systems have shown reduced performance in burn-injured patients [19,20,21,22,23,24,25]. To address these limitations, the ABA called expert panelists in 2007 to set up revised definitions for infection and sepsis, which included higher thresholds for systemic inflammatory response criteria and emphasized that burn sepsis should be presumed if the condition of the case was significantly modified [11]. However, in spite of these revisions, several trials have raised concerns regarding the robustness of those updated criteria.

### 4.3. The Mann-Salinas Criteria for Diagnosing Sepsis

Hogan et al. published a 2012 retrospective evaluation of the ABA criteria on 196 burn cases and found that a faint heart rate and temperature had a significant relationship with bacteremia [22]. However, sepsis does not imply the presence of bacteremia and, therefore, the criteria are not interesting. Mann-Salinas et al. displayed in 2013 an evaluation highlighting the uselessness of the systemic inflammation and ABA SIRS criteria in prognosticating sepsis in burn cases. The researchers put together their criteria (HR > 130, MAP < 60, base deficit < 6, temperature < 36 °C, the use of vasopressors, and serum glucose > 150 mg/dL), and these criteria were by far the best ones [15]. In spite of the efforts of the researchers and their developments, identifying and defining infection and sepsis in the burn patient population cases continues to change, impeding clinicians’ efforts to build a standard approach.

## 5. Advances in Sepsis and Infection Detection Beyond Suboptimal Criteria and Biomarkers

Recent advances in biomarker identification have centered on the use of PCR and gene expression (mRNA) profiling to improve diagnosis accuracy compared to existing methodologies. One such innovation is the FDA-approved PCR-based scoring system Septicyte (Immunexpress, Seattle, WA, USA), which utilizes proprietary biomarker signatures to detect infection in as soon as 4–6 h after hospitalization by an intensivist [26]. Preferring to identify specific pathogens, this test evaluates immunity efficacy against a suspicious infection. This approach is particularly useful for burn patients, where the practical tips regarding infection and the body’s reaction to infection frequently overlap. Differentiating between an infection-positive immune reaction and an infection-negative reaction of the body is critical for guiding appropriate therapeutic resolutions.

Regarding gene-expression-based scoring methods, tools such as the Sepsis Metascore and the FAIM3 ratio are also employed in critical care settings [26]. A 2019 independent analysis comparing these systems found that the Sepsis Metascore had the best precision in distinguishing sepsis from non-septic inflammation, with area under the curve (AUC) values of 0.80 for the Metascore, 0.69 for the FAIM3 ratio, and 0.68 for Septicyte Lab [27]. However, it is important to note that while gene expression monitoring can suggest the type of microorganism present, it does not always differentiate between colonization and active infection. This distinction is vital to limit unnecessary antibiotic use and prevent the development of antimicrobial resistance. How PCR and mRNA monitoring should be integrated into diagnostic and treatment algorithms remains a subject of ongoing investigation.

Despite the promise of PCR and mRNA profiling, as well as mass spectrometry for rapid pathogen identification, several challenges remain. One major concern is the cost, which may limit the widespread adoption of these tools in burn ICUs, particularly in resource-limited settings. Furthermore, these technologies are not yet available in all countries. Currently, mass spectrometry is widely used to identify microorganisms in patients with potential infections; while it provides extensive data covering bacteria, fungi, and viruses, there is no universally accepted standard for differentiating colonization from deep skin infections [28,29].

Accurate antimicrobial therapy relies upon the precise diagnosis of the causative infection agent. Traditionally, cultures are positive and evaluated microscopically, a process that can take several days. Mass spectrometry, particularly matrix-assisted laser desorption/ionization time-of-flight mass spectrometry (MALDI-TOF MS), offers faster alternative used for identifying bacteria, yeast, and fungi based on their microbial proteins [29]. These produce unique molecular fingerprints, or spectra, that can be referenced in a database for rapid identification. Currently, two FDA-approved databases, VITEK MSand MALDI Biotyper (BIOMIC V3 Microbiology System), are available, though many additional databases are in development [30,31].

## 6. Current Bedside Practices for Preventing and Treating Early Infection

As of 2024, bedside practices for diagnosing and treating sepsis in burn patients remain heavily reliant on clinical signs and traditional diagnostic methods. Infection continues to be the leading cause of morbidity and mortality in cases of grave burns. In contrast with classical ICUs, where hypotension, tachypnea, desaturation, and loss of consciousness may be attributed to conditions such as pulmonary embolism or myocardial infarction, these symptoms in burn patients are more frequently associated with sepsis. Therefore, infection prevention is paramount in burn units.

Strict hygienic measures are critical to preventing infection and sepsis, as burns provide an entry point for microbial pathogens. Early surgical interventions, including debridement and skin grafting, are vital components of the treatment regimen [32,33]. Additionally, the use of showers and mattresses equipped with bacterial filters has proven effective in reducing the occurrence of burn wound infections and multidrug-resistant bacterial colonization [29,30]. Another significant source of infection is fluid accumulation in dependent areas, such as the pleural space, which can lead to atelectasis and secondary pneumonia [29,30].

Topical antibacterial substances, like silver sulfadiazine and mafenide acetate, ameliorate survival rates according to recent publications in cases of grave burns [34]. These agents are known as sulfamylon creams, and the solution is no longer available in the United States of America. However, the prolonged use of these agents may contribute to bacteria no longer responding to antibiotics [35,36]. Rotating the utilization of topical agents can help maintain bacterial susceptibility to antibiotics [37]. Conversely, the use of topical antibiotics like aminoglycosides is not recommended due to the increased risk of antibiotic resistance [38,39].

At the bedside, the reality differs from the sophisticated tools discussed in earlier sections, as many of these technologies are not yet available for routine clinical practice. In rare cases, such as with very young patients who have no effective antibiotic options, expensive therapies like bacteriophage treatment may be employed and have the potential to save lives [35,36,37,38]. Additionally, other therapeutic interventions may be used in specific circumstances to address infections [40,41,42,43].

## 7. Treating Early Infections: Specific Considerations for Burn Units

In burn patients, any infection might be suspected to originate from the central venous catheter in the absence of proof to the contrary [40]. It was once standard practice to change central venous catheters every three days to reduce the risk of bloodstream infections [44], but this approach is now considered outdated. The frequency of catheter changes should instead be determined by the insertion site. For example, when the internal jugular vein is used and the puncture area is unburned, there is no reason to deviate from the protocols applied to other ICU patients. However, a femoral catheter placed in a burned area presents a significantly higher risk of infection. Critically burned patients differ from other critically ill patients in that they require a multidisciplinary approach, best delivered in dedicated burn centers. Care in these settings focuses on the specific pathophysiological challenges associated with burns, including inhalation injury, edema formation, and hypermetabolic responses.

Burn patients typically exhibit elevated core temperatures (approximately 38 °C) due to their hypermetabolic state. While minimizing pain and distress can reduce metabolic demand, completely eliminating pain is often unattainable. Similarly, controlling infection and sepsis is essential for reducing metabolic stress but remains difficult [45,46]. Because of persistent exposure to microbial products and their hypermetabolic state, burn patients often have elevated temperatures, tachycardia, and fluctuating white blood cell counts. By definition, all patients with extensive burns meet the standards for global inflammation.

The classically seen Gram-negative bugs in burn centers include *Pseudomonas aeruginosa* (74%), *Escherichia coli* (35%), *Acinetobacter baumannii* (24%), coagulase-negative staphylococci (21%), and Enterococcus species (14%) [47]. Sepsis is responsible for 50–60% of deaths in cases with grave burns [44,45,46,47]. The risk of infection and fatality is particularly elevated in young children (under four years) and elderly adults (over 55 years) compared to other age groups [43]. Biofilms—structured microbial communities encased in a self-produced extracellular polysaccharide matrix—pose a significant risk for persistent infection [44,45,46,47]. The likelihood of invasive burn wound infection relies upon factors such as the surface area and depth of the burns, the case immune situation, and the type and quantity of microbial flora colonizing the wounds [44,45,46,47,48].

**Figure 2:**
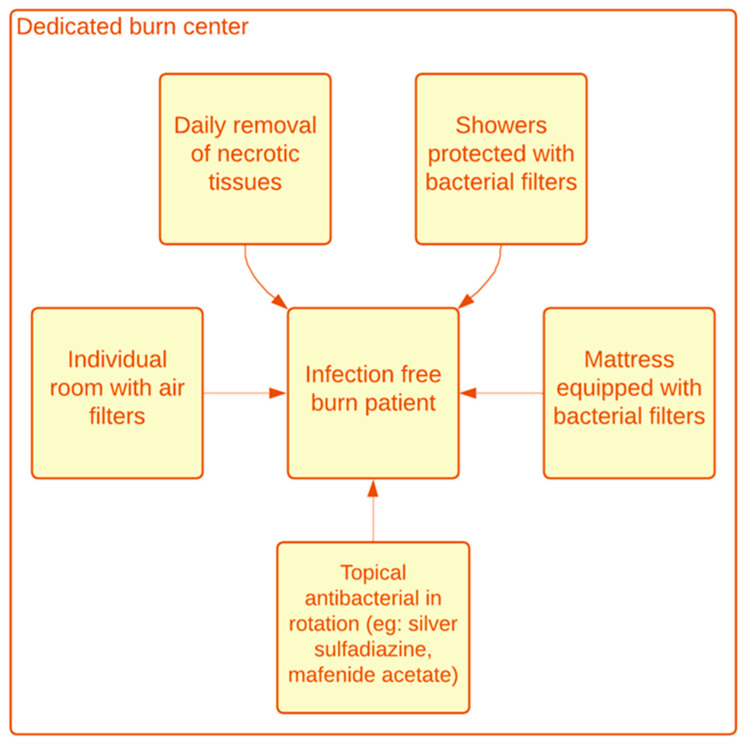
Figure summarizing prevention strategies.

In cases with grave burns, the body immune response to infection can be controlled if the burn wounds and any infections are treated effectively. However, if left untreated, this response may become uncontrolled, leading to vessel endothelial failure, slowed down wound healing, depressed immunity, and the exacerbation of SIRS [44,45,46,47,48,49,50,51]. Compared to cases with burns alone, sepsis associated with burns induced a doubling of the catabolic reaction, evaluated by isotopic methods [52]. Furthermore, disturbances in Th-17 cytokines, including IL-17 and IL-22, may slow down wound healing and contribute to burn-related sepsis [53]. Persistent hypermetabolism leads to increased levels of glycolysis, lipolysis, and proteolysis, resulting in muscle wasting and weight loss, which further impair the immune response and slow down wound healing [52,53,54]. Preventing infection in cultured skin grafts is also critical to improving outcomes [55,56,57,58]. A comprehensive approach to managing infection in severely burned patients is essential for reducing the morbidity and mortality associated with sepsis and infection. A figure summarizing prevention strategies is provided (Figure 2).

## 8. Conclusions

The early detection, diagnosis, prevention, and treatment of infection to prevent sepsis and septic shock remain significant challenges in severely burned patients. While the most recent ABA criteria, published in 2017, are currently the best available for diagnosing early infections, they still have notable limitations. For early sepsis detection, the Sepsis-3 criteria have been proven to be superior to both the ABA and Mann-Salinas criteria. The authors of the recent ABA guidelines recommend that sepsis be clinically evaluated, diagnosed, and registered in a prospective manner by a burn intensivist, although this is not always feasible.

Infection prevention remains a cornerstone of burn care, with daily baths and early surgical intervention being critical components of treatment. It is well established that daily baths significantly reduce the occurrence of burn wound infections and the development of multidrug-resistant bacteria in less severe cases. The utilization of antimicrobial topical substances was able to improve survival rates in severely burned patients. Additionally, antibiotics must be selected judiciously, and rotating antibiotics can help mitigate resistance, particularly among Gram-negative bacteria.

Despite these advances, significant gaps remain in improving the early detection, diagnosis, prevention, and treatment of infection to prevent sepsis and septic shock in severely burned patients. In current practice, burn intensivists continue to rely on clinical judgment and traditional biomarkers like CRP and PCT. The challenge lies in making these newer, revolutionary biomarkers accessible to every burn center, with cost being a major limiting factor. Furthermore, the limited number of specialized burn centers worldwide makes it difficult to conduct large-scale randomized controlled trials (RCTs), which are crucial for advancing the early detection, prevention, and treatment of infections. There is an urgent need for large international congresses on burn infections to bring together burn intensivists from around the world to initiate these essential RCTs.

## Data Availability

No new data were created or analyzed in this study. Data sharing is not applicable to this article.
